# Valsalva retinopathy in pregnancy: a case report

**DOI:** 10.1186/s13104-015-1029-8

**Published:** 2015-03-04

**Authors:** Fateme Tara, Mohammad Sharifi, Elnaz Hoseini

**Affiliations:** Obstetrics and Gynecology, Mashhad University of Medical Sciences, Mashhad, Iran; Ophthalmology, Eye Research Center, Mashhad University of Medical Sciences, Mashhad, Iran; Womens health research center, school of medicine, Mashhad university of medical sciences, Mashhad, Iran

**Keywords:** Retinopathy, Valsalva, Pregnancy

## Abstract

**Background:**

Valsalva retinopathy is a known medical condition which happens after valsalva maneuver. The aim of this study was to report a case of young asian pregnant female presented with acute visual loss due to the valsalva retinopathy, followed by spontaneous visual recovery*.*

**Case presentation:**

A case of 23 years-old pregnant Asian female with 31 weeks of gestation presented with history of a sudden decrease in vision in her left eye from seven hours prior to the visit. Based on clinical findings, laboratory test and imaging studies, the diagnosis of Valsalva retinopathy was confirmed. Subsequently, her normal vision was returned during three months with no intervention.

**Conclusion:**

Pregnancy is a risk factor for Valsalva retinopathy. Valsalva retinopathy generally has a good prognosis. Vision usually returns to normal over a short period of time and requires only observation.

## Background

Valsalva retinopathy was first described in 1972 by Thomas Duane as “a particular form of retinopathy, pre-retinal and hemorrhagic in nature, secondary to a sudden increase in intrathoracic pressure.” Immediately after a Valsalva maneuver, there is a sudden increase in intraocular venous pressure causes retinal capillaries to rupture spontaneously [1].

The patient often gives a history of a recent strenuous physical activity, which may increase the intra-thoracic pressure. Pregnancy is known to be a risk factor for Valsalva retinopathy [2]. We, therefore, report valsalva retinopathy in a young healthy pregnant woman with spontaneous resolution with no sequelae.

## Case presentation

**Figure 1:**
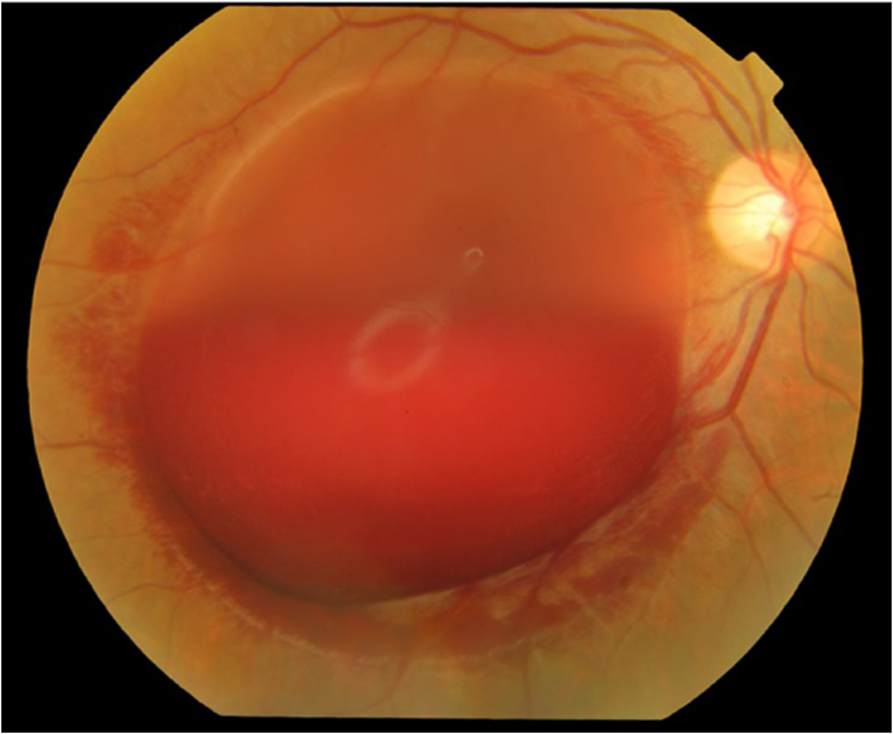
**Large pre retinal macular hemorrhage in valsalva retinopathy.**

**Figure 2:**
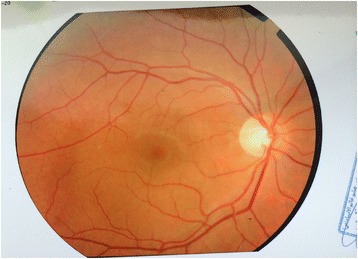
**Complete absorption of macular hemorrhage after three months in valsava retinopathy.**

A 23 years old pregnant Asian female with 31 weeks of gestation presented with a history of a sudden decrease in vision in her left eye from seven hours prior to the initial visit. Her clinical problems were initiated after severe retching and vomiting due to the ingestion of ferrous sulfate tablets. Her previous medical history was unremarkable. Ophthalmic examination showed best corrected visual acuity of 20/20 in the right eye and count finger in 2 meters in the left eye. Pupillary response, eye movement and anterior segment evaluations were normal. Fundus examination revealed large (10–15 disc diameters) pre-retinal hemorrhage located in the macula (Figure 1). Blood pressure (BP), complete blood count (CBC), coagulation profiles and fasting blood glucose (FBS) were within the normal ranges. Based on the clinical findings and laboratory studies, the diagnosis of Valsalva retinopathy was confirmed. Conservative management including rest position, observation of vital signs and avoidance of strenuous activities were recommended. Finally, she underwent normal vaginal delivery (NVD) without any complications. The vision of the patient returned to 20/20 and hemorrhage was resorbed completely during three months after the delivery (Figure 2).

## Discussion

Valsalva retinopathy usually occurs in healthy adults and has been reported following coughing, weight lifting, vomiting, jumping, end-stage of labor, episodes of constipation, vomiting and lifting in pregnant women [1,3].

Pregnancy exerts multiple hormonal, metabolic, hematological and immunological alterations in the mother that may represent risk factors for Valsalva retinopathy.

Elevation of intra-abdominal pressure during pregnancy, with a further increase during labor, leads to a considerable elevation in intravenous pressure. This may increase the potential for retinal hemorrhages following a Valsalva maneuver [2].

Moreover, hematological alterations during the pregnancy such as thrombocytopenia add to the risk of Valsalva retinopathy in pregnancy [2]. In such clinical scenarios, it is important to rule out all systemic diseases that may result in retinal hemorrhages such as diabetes, hypertension, sickle cell disease, anemia, coagulopathy, blood dyscrasias and previous ocular vein occlusions [2].

The prognosis for patients diagnosed with merely Valsalva retinopathy is generally good and the condition requires only close observations. Vision usually returns to normal over a short period of time, from weeks to months [1]. Some patients may have a poor visual outcome, which has been attributed to retinal pigmentary changes in the macula [4].

An yttrium aluminum garnet (YAG) laser has been employed in selective cases to disperse the pre-retinal hemorrhages and speed up the resolution [5].

Whether vaginal delivery poses a risk of recurrence or exacerbation of the hemorrhage is not fully understood [3]. A review of the literature showed no recurrence of retinopathy following spontaneous vaginal delivery [3,4]. The case of the present study had normal vaginal delivery which was recommended by her obstetrician. Epidural anesthesia accompanied with adequate sedation was applied for the decrease of pain and strenuous activity during labor.

## Conclusion

Valsalva retinopathy is a self-limited condition. Pregnancy is one of the known risk factor for this condition. Most patients are managed conservatively with good outcome.

## Consent statement

Written informed consent was obtained from the patient for publication of this case report and any accompanying images. A copy of the written consent is available for review by the Editor-in-Chief of this journal.

## Abbreviation

YAG: Yttrium aluminum garnet
